# DECISION-MAKING CAPACITY ASSESSMENTS FOR VULNERABLE ADULTS: LESSONS FOR SOCIAL WORK COMPETENCY

**DOI:** 10.1093/geroni/igad104.3258

**Published:** 2023-12-21

**Authors:** Adria Navarro, Eppie Leishman

**Affiliations:** University of Southern California, Los Angeles, California, United States; The University of York, York, England, United Kingdom

## Abstract

Social work approaches to the assessment of decision-making capacity (DMC) assessment have largely been informed by literature from 25 years ago. Evidence-based practices include the ACED short version and the IDA-CA 3.0. When considering this specific practice by social workers there is the UK policy supporting competent practice through legislation, the Mental Capacity Act (2017) of England and Wales. Nine practitioners practicing through guidance presented by this policy were interviewed to learn what may support practices in the United States. Findings point to the importance of foundational values and efforts to optimize the vulnerable adult’s performance. Practical methods used in the assessment are gleaned, along with the training that has informed their work. Training advice included didactic, case review, and role-playing of increasingly complex cases. Experience working with diverse populations of vulnerable adults was also highlighted. This research strives to inform steps that enhance capacity within health and social care settings to adequately assess DMC. Understanding decision-making ability aids in preventing both paternalism and the insufficiency of support and/or protection.

